# Linking leptin in GCF with tooth movement among Indians

**DOI:** 10.6026/97320630018572

**Published:** 2022-06-30

**Authors:** Soumyadev Satpathy, Vidyesh Nadkerny, Shaivi Sharma, Ayyagari Viresh Goutameshwar, Sidharth Joshi, Vibhuti

**Affiliations:** 1 Department of Dentistry, Fakir Mohan Medical College, Balasore, Odisha 756019, India; 2 Department of Orthodontics, Daswani Dental College & Research Centre, Kota, 324001, Rajasthan, India; 3 Dept of orthodontics, Bhabha dental college Bhopal - 462001, Madhya Pradesh, India; 4 Department of Conservative Dentistry and Endodontics, JSS Dental College and Hospital Mysuru, Mysuru - 570004, Karnataka, India; 5 Department of Periodontology, Aditya Dental College, Beed, 431122, Maharastra, India; 6 Department of Conservative Dentistry & Endodontics, Maharishi Markendeshwar College of Dental Sciences & Research, Mullana, Ambala - 133203 Haryana, India

**Keywords:** GCF, leptin concentration, orthodontic force, tooth movement

## Abstract

It is of interest to evaluate the association of orthodontic tooth movement with concentration of leptin in Gingival Crevicular Fluid (GCF).Thirty orthodontic patients of both genders with equal sample size were included for the present study. Concentration of leptin was assessed at baseline (T0), 1 hr after application of force (T1), 24 hours later (T2), 7 days after application of force (T3), and 1 month after application of orthodontic force (T4). Using strips of filter paper, GCF was collected from the gingival sulcus on distal aspect of the right maxillary canine. Distalized tooth movement was evaluated by measuring the difference on dental casts, at baseline and one month after force application. One-way ANOVA with Bonferroni correction and Pearson's correlation test were used to analyze the data. Average concentration of leptin in GCF raises from baseline (T0) to 1 hours after application of force (T1), then increased to peak after 24 hours (T2), and declined to a minimum value after 7 days (T3) and again raises after 1 month (T4), closer to the base line value (T0), and this was statistically significant (P < 0.05). There was significant association of the overall average concentration of leptin to degree of tooth movement (correlation coefficient = 0.625). There was a biphasic change in GCF leptin concentration during one cycle of orthodontic force application, thus, a significant association between rates of tooth movement with GCF leptin concentration is noted.

## Background:

Orthodontic tooth movement is a process of physiological bone remodeling with minor reversible injury to the periodontium in response to mechanical force applied by an orthodontic appliance [1-3]. It has been observed that, during orthodontic tooth movement, there will be significantly raise in numerous cytokines such as; interleukin 1-beta,interleukin-6, pentraxin -3, B2 microglobulin, tumor necrosis factor α, matrix metallo-proteinases, and enzymes such as, lactate dehydrogenase, and alkaline phosphatase [2,4- 7]. Leptin levels during infection and inflammation designates that leptin has role in the host defense mechanisms and immune response [2]. Leptin which is found in the Gingival Crevicular Fluid (GCF), has higher concentrations in subjects with healthy gingival tissues as compared to subjects with periodontal disease [2,8, 9]. Previous several studies have demonstrated the role of GCF leptin activity in development of periodontal disease and its presence in marginally inflamed and in healthy gingivae. Leptin has direct role in bone formation by enhancing osteoblast proliferation and differentiation. It maintains bone levels by protecting the host from infection and inflammation. During orthodontic tooth movement with stress-strain distribution, there will be a remodeling process (resorption and apposition) takes place in periodontal tissues, along with it. There will be local inflammation-like reactions with damage-repair process, including high vascular activity with macrophages and numerous leukocytes and participation of immune system [2]. Therefore, it is of interest to evaluate the association of orthodontic tooth movement with concentration of leptin in Gingival Crevicular Fluid (GCF).

## Materials and methods:

Thirty orthodontic patients of both genders with equal sample size were included for the present observational prospective study. This study was conducted in the department of Orthodontics and dentofacial orthopedics, after obtaining approval from institutional ethics committee. This study was done form April 2017 to August 2019. Informed consent was obtained from all the participants prior to the study. Sample size was calculated according to Dilsiz et al. (year) study with power (1-β error) = 0.8, α error probability = 0.05 and effect size f = 0.28 [2]. Participant’s average age was 21.5+_1.87 and in the age ranges of 19-25 years. Inclusion criteria was; patients with Angle's class I malocclusion with ANB angle 2° ±2 and class I skeletal base, maxillary crowding more than 5 mm which requires canine distalisation following extraction of all 1st premolars in healthy patients. Exclusion criteria were subjects with presence of oral habits and subjects not willing to participate in the study. Fixed orthodontic alignment was initiated with initial archwire of 014-inch NiTi. Two weeks after the extraction of all 1st premolars, distalisation of canine was initiated with active lace-backs, from canine to 1st molar using 0.009" stainless steel wire. The force application was made standardized by giving an equivalent number of turns (four turns) for each participant. Trans-palatal arch on 1st molar was used as an anchorage. Using strips of filter paper (Periopapers, Ora flow Inc, New York,). GCF was collected from the gingival sulcus on distal aspect of the right maxillary canine. Patients were instructed to maintain good oral hygiene to prevent gingival inflammation throughout the period of the study. This was confirmed by evaluating the bleeding on probing and probing depth at each time before collecting sample. Blood and saliva contaminated samples were discarded. The GCF sample was collected in morning time at each recall visit. Concentration of leptin was assessed at baseline (T0), 1 hour after application of force (T1), 24 hours later (T2), 7 days after application of force (T3), and 1 month after application of orthodontic force (T4). Base line acts as control.

### Leptin analysis:

Each filter paper strip was eluted twice with 100 µL Hank's balanced salt solution including 0.5% bovine serum albumin by centrifugation (3000 x g; 4°C) for 15 minutes. Concentration of leptin was evaluated by commercially available enzyme-linked immunosorbent assay. The assays were carried out according to the instructions from manufacturer. For leptin assays, high-sensitivity kits (Bio Source International Inc, Camarillo, Calif) were used to quantitatively detect low levels of leptin. 

### Measurement of the rate of tooth movement:

Casts were made from alginate impression at baseline (T0) and after one month after application of force (T4). Rate of tooth movement was calculated as the amount of distal movement of the maxillary canine at the end of one month. Amount of canine movement was calculated using Vernier caliper at baseline and after 1 month on cast. Difference between these two values was taken as the rate of tooth movement. All the procedure was done by trained single operator. Repetition of the procedure was done at a time period of 1 week on dental casts and method.

### Statistical analysis:

The obtained data was tabulated and statistically analyzed using SPSS software version 21, SPSS, Chicago with "t" test to calculate mean GCF leptin concentration and rate of tooth movement. Mean leptin concentration at the five time points was calculated wit h one-way ANOVA with Bonferroni correction. Kolmogorov Smirnov test was used to check normality of distribution of data (Table 1 - see PDF). Using Pearson's correlation coefficient, mean degree of tooth movement was calculated, and it was associated to mean concentration of leptin.

## Result:

Table 2(see PDF) shows mean GCF leptin concentration at various time points. Average leptin concentration in GCF raises from baseline (T0, 316.475 picograms/micro liter) to 1 hours after application of force (T1, 394.815 pg/µL), then increased to peak at after 24 hours (T2, 943.975 pg/µL), and declined to a minimum value after 7 days (T3, 30.21 pg/µL) and again raises after 1 month (T4, 245.985 pg/µL), closer to the base line value (T0), and this was statistically significant (P < 0.05). Table 3(see PDF shows comparison of average GCF leptin concentration at various time points. It was statistically significant (P<0.005) except for value at T2 which was not statistically significant when compared with values at T0 and T1.

This could be due to raised 95% confidence interval at T2. Mean concentration of leptin and degree of tooth movement did not have any statistically considerable gender variation (P > 0.05) (Table 4 - see PDF). There was significant association of the overall mean concentration of leptin to degree of tooth movement (correlation coefficient = 0.625) Table 5(see PDF). Figure 1 shows mean and standard deviation of GCF Leptin Concentration at T0, T1, T2, T3 and T4. Values at T4 were somewhat lower than the concentration at baseline T0. This oblique that leptin levels could reach baseline values within a month following a single orthodontic activation.

## Discussion:

Orthodontics is a branch of dentistry that deals with correction malocclusion. From past several decades, numbers of patients undergoing orthodontic treatment have increased enormously. During orthodontic tooth movement, mechanical stress from orthodontic appliances is believed to stimulate the periodontal ligament (PDL) cells to form biologically active substances, resulting into bone remodeling and tooth movement. Since leptin has role in bone remodeling action, this study was done to evaluate the role of leptin level on degree of tooth movement. Dilsiz et al. evaluated concentration of leptin in GCF before and after application of orthodontic force and concluded that concentration of leptin in GCF is decreased by orthodontic tooth movement; which is contradictory to our results [2]. Srenivasan et al. evaluated the leptin concentration in GCF and assessed its correlation with rate of orthodontic tooth movement. They concluded that there was positive correlation among tooth movement and GCF leptin concentration [8]. These results are in accordance to our findings. Jain et al. evaluated the level of Leptin in GCF during Orthodontic tooth Movement from baseline to 7 days after tooth force application and found that Leptin may be one of the mediators associated with orthodontic tooth movement [9]. This is in agreement to our results. Jayachandran et al. compared the salivary leptin concentration among overweight and normal weight persons and rate of tooth movement. They observed decreased degree of tooth movement in obese individuals and salivary leptin levels and rate of tooth movement in both groups was in positive proportional [10]. However Von Bremen et al. stated that duration of orthodontic treatment was superior in obese persons [11]. Such persons have a better serum leptin levels and improved bone mineral density. Many studies indicated that higher concentration of the intermediaries in GCF following application of orthodontic force and this increase in leptin levels was accredited to inflammatory reaction of periodontal ligament to the orthodontic force [8,9,10]. In present study, we have evaluated the concentration of leptin in GCF during one cycle of orthodontic force application. There were 2 phase changes in leptin concentration, with a peak at one day (T2) after application of force and drops down after one month (T3), which was less significant than the base line (T0) rate. The primary raise in leptin level in the GCF at T1, T2 could be evaluated to rise in secretion of any inflammatory mediator after application of force. The initial raise in leptin concentration was followed by noticeable diminish after one week of application of force which could be due to raise in leptin receptors in the periodontal ligament following the initial raise in leptin level which, in turn, could have resulted in formation of more leptin -leptin receptor complexes, leading to a reduced levels of leptin in the GCF resulting in reduction in GCF leptin levels after 1 week. We found positive correlation with leptin level and tooth movement. The positive association could be accredited to its osseo-regulatory and remodeling actions [8]. Interleukin 1 beta was interrelated to the degree of tooth movement, among the diverse biomarkers estimated for orthodontic tooth movement. There was a affirmative association between interleukin 1 beta concentration and the degree of tooth movement [8, 12]. Leptin is found to control the interleukin system, thus optimistic relationship of GCF leptin concentration to degree of tooth movement [13]. In the present study, concentration of leptin was in positive proportional to degree of tooth movement when assessed for one month period. This can be considered along with other parameters in enhancing the rate of orthodontic tooth movement. Limitation of the present study is shorter duration and smaller sample size. Additional studies at the molecular stage during orthodontic tooth movement are necessary to establish the accurate biological role of leptin in orthodontic tooth movement.

## Conclusion:

Leptin concentration in GCF is positively associated with degree of tooth movement. Thus, there was a biphasic changes in GCF leptin concentration during one cycle of orthodontic force application form baseline to one month postoperatively.
